# Crystal structure and photoluminescent properties of bis­(4′-chloro-2,2′:6′,2′′-terpyrid­yl)cobalt(II) dichloride tetra­hydrate

**DOI:** 10.1107/S205698902000287X

**Published:** 2020-03-05

**Authors:** B. Thippeswamy, P. A. Suchetan, K. M. Mahadevan, H. Nagabhushana, G. R. Vijayakumar

**Affiliations:** a Department of Chemistry, University College of Science, Tumkur University, Tumkur, Karnataka 572 103, India; b Department of Chemistry, Government Science College, Chitradurga, 577501, India; c Department of Chemistry, Kuvempu University, P. G. Centre, Kadur-577548, India; d Prof. C. N. R Rao Centre for Advanced Materials Research, Tumkur University, Tumkur-572 103, India

**Keywords:** crystal structure, 2,2′,6′,2′′-terpyridine, photoluminescence, SEM, TG–DTA

## Abstract

In the title hydrated complex, [Co(C_15_H_10_ClN_3_)_2_]Cl_2_·4H_2_O, the complete dication is generated by  symmetry. The CoN_6_ moiety shows distortion from regular octa­hedral geometry with the *trans* bond angles of two N—Co—N units being 160.62 (9)°. In the crystal, O—H⋯Cl and C—H⋯O inter­actions link the components into (001) sheets. The title compound exhibits blue-light emission, as indicated by photoluminescence data, and a HOMO–LUMO energy separation of 2.23 eV was obtained from its diffuse reflectance spectrum.

## Abstract 2

In the title hydrated complex, [Co(C_15_H_10_ClN_3_)_2_]Cl_2_·4H_2_O, the complete dication is generated by 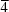 symmetry. In the crystal, O—H⋯Cl and C—H⋯O hydrogen bonds link the components into (001) sheets. The complex exhibits blue-light emission.

## Chemical context   

Since the pioneering work of Tang *et al.* (1987 ▸), there has been increasing inter­est in chelating organic compounds being employed as charge-transporting materials in electronic devices such as OLEDs. Transition-metal complexes are promising candidates for use as hole-transporting materials as the metal ions can assume variable oxidation states and are found to exhibit low kinetic barriers for self-exchange reactions (Marcus, 1965 ▸).

As 2,2′-bi­pyridine (bpy) is reported to show both σ-donor and π-acceptor capabilities, disubstituted 4,4′-, 5,5′- and 6,6′-derivatives of bpy have been widely employed in supra­molecular and coordination chemistry (Kaes *et al.*, 2000 ▸; Williams *et al.*, 2002 ▸). Materials incorporating pyridine have also been shown to perform well in electron-transporting layers in OLEDs because of their high electron mobility (Ichikawa *et al.*, 2010 ▸).

Single-layer device structures that make use of Ru^II^ complexes involving bi­pyridine and its derivatives not only show the potential to transport both holes and electrons but also exhibit luminescent properties (Rudmann & Rubner, 2001 ▸; Gao & Bard, 2000 ▸). Reports of the application of cyclo­metalated Ir^III^ complexes in vapour-deposited OLEDs both as efficient emissive and charge-transporting materials (Adamovich *et al.*, 2003 ▸; Grushin *et al.*, 2001 ▸) and the luminescent properties of a distorted octa­hedral Ni^II^ complex with 5,5′-dimethyl-2,2′-bi­pyridine have been published (Abedi *et al.*, 2015 ▸). The synthesis and a study of the thermal and luminescent properties of *d* ^8^ transition-metal complexes with the incorporation of substituted 2,2′;6′,2′′-terpyridine ligands were described by Momeni *et al.* (2017 ▸).

As an extension of such studies, we now report the synthesis, structure, spectroscopic characterization and thermal behaviour of the title complex, (I).
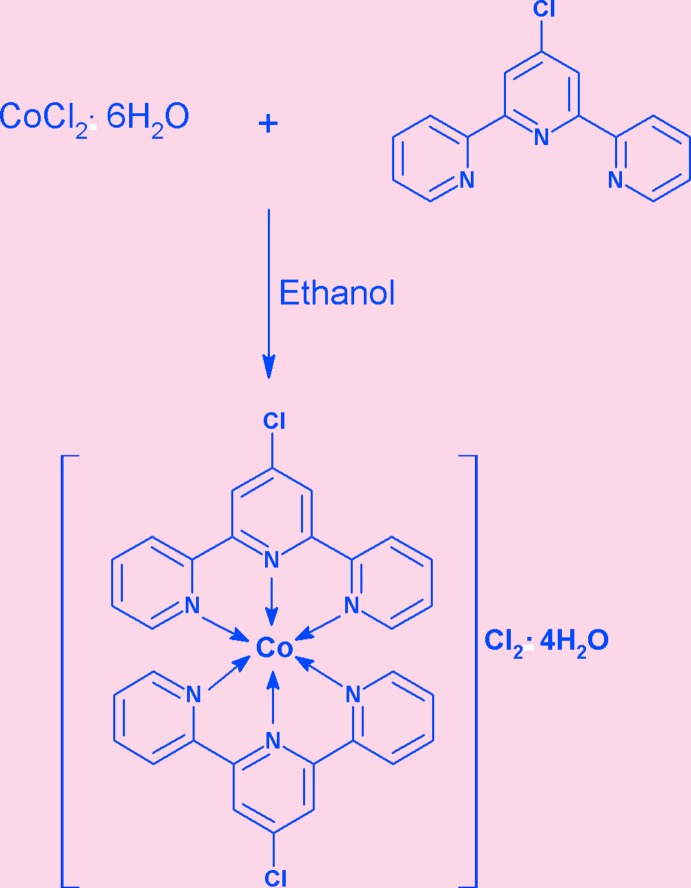


## Structural commentary   

**Figure 1:**
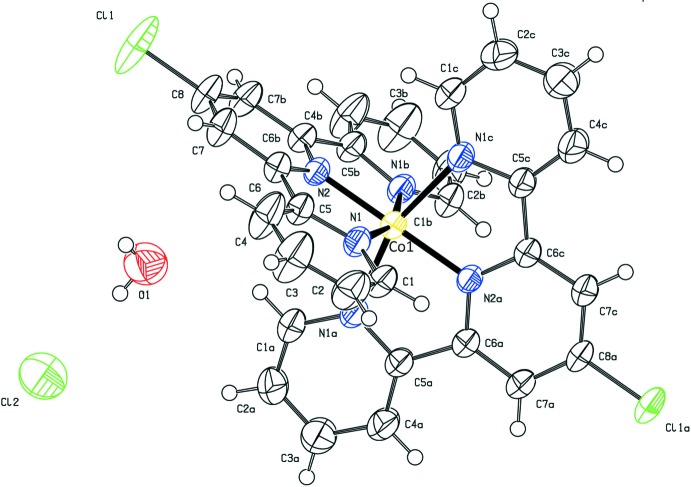
The mol­ecular structure of (I) with displacement ellipsoids drawn at the 50% probability level. The complete cation of the complex is generated by applying the symmetry operations (*a*) −*y* + 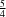, *x* + 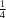, −*z* + 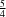, (*b*) −*x* + 1, −*y* + 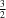, *z* and (*c*) *y* − 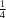, −*x* + 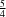, −*z* + 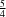.

The [Co(C_15_H_10_N_3_)_2_Cl_2_]^2+^ cation in (I) is symmetric (the metal atom lies on a special position with 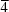 site symmetry; atoms N2, C8 and Cl1 lie on a crystallographic twofold axis), thus the asymmetric unit contains half of the ligand coordinated to the cobalt ion, one water mol­ecule of crystallization (O atom site symmetry 1) and half of a chloride counter-ion (site symmetry 2) (Fig. 1 ▸). The complex shows distortion from an ideal octa­hedral geometry for the metal ion with two N1—Co1—N1 bond angles being 160.62 (9)°. However, the N2—Co1—N2 bond angle is 180°, as it lies on the rotoinversion axis. The coordinated ligand is almost planar with the r.m.s. deviation of all the non-hydrogen atoms being 0.025 Å. Moreover, the dihedral angle between the ligands is 90.0°, as constrained by the presence of the rotoinversion axis.

## Supra­molecular features   

**Figure 2:**
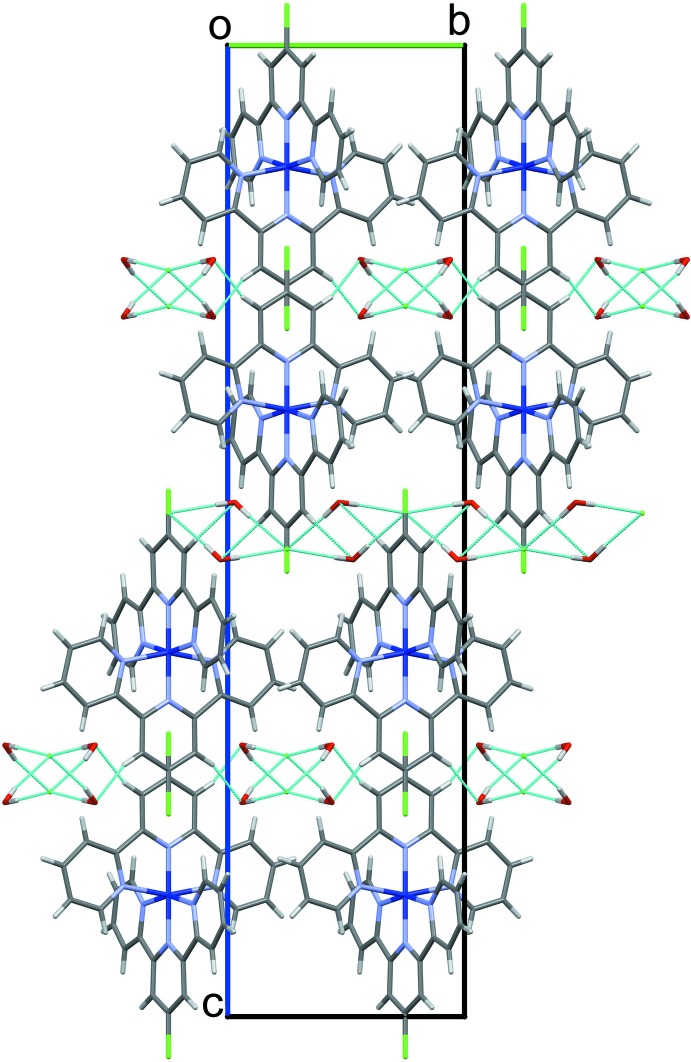
A partial view of the crystal packing of (I) viewed down [100]. Hydrogen bonds are shown as thin blue lines.

The unit cell of (I) contains four cations, which are electrically balanced by eight chloride ions along with sixteen water mol­ecules of crystallization (Fig. 2 ▸). In the crystal structure, two pairs of O—H⋯Cl hydrogen bonds between water mol­ecules and chloride ions [O2—H2*O*1⋯Cl2 and O2—H1*O*1⋯Cl2] link the components into infinite (001) sheets (Table 1 ▸).

## Thermal and photoluminescence studies   

Thermogravimetry (TG) and differential thermal analysis (DTA) on (I) show progressive decomposition in several steps. The first mass loss (obs. 10.0%, calc. 9.8% over the temperature range 60–140°C) is attributed to the loss of the water mol­ecules of crystallization, accompanied by endotherms at 78 and 134°C. The second mass loss over the temperature range 200–310°C accompanied by a DTA peak at 306°C is probably due to the decomposition of one ligand with an estimated mass loss of 36.1% (calcd. mass loss 36.2%). Powder XRD of the final residue after heating to 800°C indicated the presence of cobalt oxy hydroxide, CoO(OH) and Co_3_O_4_ (Sulikowska *et al.*, 2000 ▸).

**Figure 3:**
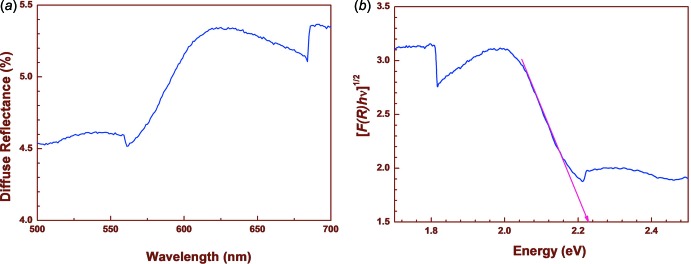
(*a*) Diffuse reflectance spectrum of (I) (*b*) Plot of [*F(R*_∞_ *)hν*]^1/2^ *versus* energy for (I)

The diffuse reflectance (DR) spectrum of (I) was scanned in the wavelength range 200–1100 nm and an absorption band appeared in the visible region as shown in Fig. 3 ▸ *a*. A prominent peak with a diffuse reflectance percentage of 5.4 is observed at 640 nm. The Kubelka–Munk function (Harry, 1976 ▸) (Fig. 3 ▸ *b*) was used in order to determine the HOMO–LUMO gap for (I): the band gap energy obtained from the plot was found to be 2.23 eV (Morales *et al.*, 2007 ▸).

**Figure 4:**
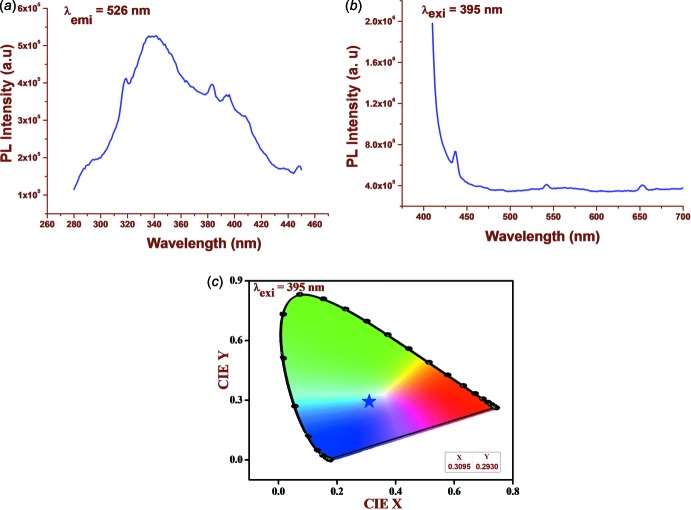
Photoluminescence spectra of (I); (*a*) excitation spectrum (*b*) emission spectrum (*c*) CIE graph

The excitation and emission spectra of (I) recorded at room temperature are shown in Fig. 4 ▸ *a* and *b*. The excitation spectrum shows features at 318, 339, 382 and 395 nm. From the emission spectrum, three well-defined peaks at 436, 541 and 653 nm are apparent for (I). The determination of chromaticity co-ordinates [Publication CIE No 15.2 (1986 ▸) and 17.4 (1987 ▸)] was carried out at an excitation wavelength of 395 nm. The estimated CIE values for the probable excitation are incorporated in the left corner of Fig. 4 ▸ *c*. The colour of emission for the highlighted phosphor is indicated in the chromaticity diagram by the solid circle sign (star), which indicates that the emission colour is blue.

## Database survey   

A search of the Cambridge Structural Database gave 90 matches for crystal structures containing the 4′-chloro-2,2′;6′,2′′-terpyridine (*L*) ligand. Closely related complexes to (I) with a pair of chelating *L* ligands generating an *M*N_6_ coordination sphere include the nickel and iron complexes [Ni(l-κ^3^ *N*,*N*′,*N*′′)_2_]Cl_2_·3H_2_O (CCDC refcode HIVPUY; Huang *et al.*, 2008 ▸) and [Fe(l-κ^3^ *N*,*N*′,*N*′′)_2_]Cl_2_·4H_2_O (HIVQEJ; Huang *et al.*, 2008 ▸); the latter complex is isostructural with (I). The structure of [Ru(l-κ^3^ *N*,*N*′,*N*′′)_2_]Cl_2_·2H_2_O (PAYMOT; Wang *et al.*, 2012 ▸) has also been described. The dihedral angles between the *L* ligands in HIVPUY, HIVQEJ and PAYMOT are 94.9 (3), 86.1 (3) and 87.0 (3)°, respectively. The crystals of both HIVPUY and HIVQEJ display three-dimensional networks arising from O—H⋯Cl and C—H⋯O inter­actions. In PAYMOT, the cations, anions and water mol­ecules are linked into a three-dimensional network by C—H⋯Cl, C—H⋯O and O—H⋯Cl hydrogen bonds.

## Synthesis and crystallization   

A solution of 4′-chloro-2,2′;6′,2′′-terpyridine (**2**) (0.535 g, 2.00 mmol) in 3 ml of ethanol was stirred at 333 K for about 30 min and an aqueous solution of cobalt(II) chloride hexa­hydrate (**1**) (0.2379 g, 1.00 mmol) dissolved in 2 ml of water was added slowly and the resulting solution was refluxed for one h. The brown solution obtained was subjected to slow evaporation at room temperature and was finally triturated with toluene to recover the powdered form of the title complex. The solid product was then kept in a desiccator in order to achieve constant weight (yield 0.584 g; 87.8%).

The product was recrystallized from a mixed methanol–aceto­nitrile (1:9) solvent system and brown prisms of (I) were obtained. IR (KBr, cm^−1^): 3039 (CH aromatic), 1595 (C=N aromatic), 1416–1554 (C=C aromatic), 491 and 409 (Co—N symmetric and asymmetric bending, respectively). The broad band centred near 3423 cm^−1^ can be ascribed to ν(O—H) vibrations.

Simultaneous TG/DTA measurements were carried out using a Perkin–Elmer Diamond TG/DTA analyser. A Perkin–Elmer Lambda-35 UV-visible spectrophotometer and Moriba spectrofluorimeter equipped with a 450 W xenon lamp as an excitation source were used to obtain the diffuse reflectance and photoluminescence spectra, respectively.

## Refinement   

Crystal data, data collection and structure refinement details are summarized in Table 2 ▸. The oxygen-bound H atoms were located from difference-Fourier maps and refined as riding: O—H = 0.82 (2) Å. The carbon-bound H atoms were placed in calculated positions (C—H = 0.93 Å) and were included in the refinement in the riding-model approximation, with *U*_iso_(H) set to 1.2*U*_eq_(C).

## Supplementary Material

Crystal structure: contains datablock(s) I. DOI: 10.1107/S205698902000287X/hb7878sup1.cif

Structure factors: contains datablock(s) I. DOI: 10.1107/S205698902000287X/hb7878Isup2.hkl

(a) SEM image (b) EDS spectrum of the complex (3) and elemental composition of the complex (inset table). DOI: 10.1107/S205698902000287X/hb7878sup3.docx

CCDC reference: 1914486

Additional supporting information:  crystallographic information; 3D view; checkCIF report

## Figures and Tables

**Table 1:** Hydrogen-bond geometry (Å, °)

*D*—H⋯*A*	*D*—H	H⋯*A*	*D*⋯*A*	*D*—H⋯*A*
O1—H2*O*1⋯Cl2	0.82	2.35	3.1735	177
O1—H1*O*1⋯Cl2^i^	0.84	2.43	3.2607	170
C7—H7⋯O1^ii^	0.93	2.44	3.334 (4)	161

Symmetry codes: (i) 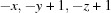; (ii) 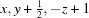.

**Table 2:** Experimental details

Crystal data	
Chemical formula	[Co(C_15_H_10_ClN_3_)_2_]Cl_2_·4H_2_O
*M*_r_	737.31
Crystal system, space group	Tetragonal, *I*4_1_/*a*
Temperature (K)	296
*a*, *c* (Å)	9.2846 (7), 38.069 (4)
*V* (Å^3^)	3281.7 (6)
*Z*	4
Radiation type	Mo *K*α
μ (mm^−1^)	0.89
Crystal size (mm)	0.35 × 0.35 × 0.30

Data collection	
Diffractometer	Bruker APEXII CCD area
Absorption correction	Multi-scan (*SADABS*; Bruker, 2009 ▸)
*T*_min_, *T*_max_	0.739, 0.765
No. of measured, independent and observed [*I* > 2σ(*I*)] reflections	12778, 2054, 1628
*R*_int_	0.031
(sin θ/λ)_max_ (Å^−1^)	0.669

Refinement	
*R*[*F* ^2^ > 2σ(*F* ^2^)], *wR*(*F* ^2^), *S*	0.044, 0.127, 1.08
No. of reflections	2054
No. of parameters	112
No. of restraints	2
H-atom treatment	H atoms treated by a mixture of independent and constrained refinement
Δρ_max_, Δρ_min_ (e Å^−3^)	0.69, −0.41

Computer programs: *APEX2* (Bruker, 2009 ▸), *SAINT-Plus* (Bruker, 2009 ▸), *SHELXT2016/4* (Sheldrick, 2015*a* ▸), *SHELXL2016/4* (Sheldrick, 2015*b* ▸), *Mercury* (Macrae *et al.*, 2020 ▸).

## supplementary crystallographic information

### Crystal data

**Table:**

[Co(C_15_H_10_ClN_3_)_2_]Cl_2_·4H_2_O	Prism
*M**_r_* = 737.31	*D*_x_ = 1.492 Mg m^−^^3^
Tetragonal, *I*4_1_/*a*	Mo *K*α radiation, λ = 0.71073 Å
Hall symbol: -I 4ad	Cell parameters from 143 reflections
*a* = 9.2846 (7) Å	θ = 2.1–28.4°
*c* = 38.069 (4) Å	µ = 0.89 mm^−^^1^
*V* = 3281.7 (6) Å^3^	*T* = 296 K
*Z* = 4	Prism, brown
*F*(000) = 1508	0.35 × 0.35 × 0.30 mm

### Data collection

**Table:**

Bruker APEXII CCD area diffractometer	2054 independent reflections
Radiation source: fine-focus sealed tube	1628 reflections with *I* > 2σ(*I*)
Graphite monochromator	*R*_int_ = 0.031
phi and φ scans	θ_max_ = 28.4°, θ_min_ = 2.1°
Absorption correction: multi-scan (SADABS; Bruker, 2009)	*h* = −10→12
*T*_min_ = 0.739, *T*_max_ = 0.765	*k* = −11→12
12778 measured reflections	*l* = −50→50

### Refinement

**Table:**

Refinement on *F*^2^	Primary atom site location: dual
Least-squares matrix: full	Hydrogen site location: mixed
*R*[*F*^2^ > 2σ(*F*^2^)] = 0.044	H atoms treated by a mixture of independent and constrained refinement
*wR*(*F*^2^) = 0.127	*w* = 1/[σ^2^(*F*_o_^2^) + (0.0659*P*)^2^ + 2.6119*P*] where *P* = (*F*_o_^2^ + 2*F*_c_^2^)/3
*S* = 1.08	(Δ/σ)_max_ < 0.001
2054 reflections	Δρ_max_ = 0.69 e Å^−^^3^
112 parameters	Δρ_min_ = −0.41 e Å^−^^3^
2 restraints	

### Special details

**Table:**

Geometry. All esds (except the esd in the dihedral angle between two l.s. planes) are estimated using the full covariance matrix. The cell esds are taken into account individually in the estimation of esds in distances, angles and torsion angles; correlations between esds in cell parameters are only used when they are defined by crystal symmetry. An approximate (isotropic) treatment of cell esds is used for estimating esds involving l.s. planes.

### Fractional atomic coordinates and isotropic or equivalent isotropic displacement parameters (Å^2^)

**Table:**

	*x*	*y*	*z*	*U*_iso_*/*U*_eq_	
Co1	0.500000	0.750000	0.625000	0.03266 (18)	
Cl1	0.500000	0.750000	0.45878 (2)	0.0973 (5)	
Cl2	0.000000	0.250000	0.51848 (4)	0.0902 (4)	
N2	0.500000	0.750000	0.57542 (6)	0.0327 (5)	
N1	0.3088 (2)	0.8560 (2)	0.61590 (4)	0.0374 (4)	
O1	0.1904 (3)	0.5302 (3)	0.52954 (7)	0.0799 (7)	
C6	0.3905 (2)	0.8121 (2)	0.55799 (5)	0.0368 (4)	
C5	0.2786 (2)	0.8709 (2)	0.58127 (5)	0.0396 (5)	
C1	0.2131 (3)	0.9052 (3)	0.63940 (6)	0.0463 (5)	
H1	0.233771	0.896268	0.663200	0.056*	
C7	0.3886 (3)	0.8165 (3)	0.52162 (5)	0.0488 (6)	
H7	0.314890	0.862669	0.509485	0.059*	
C8	0.500000	0.750000	0.50418 (8)	0.0516 (9)	
C2	0.0868 (3)	0.9678 (4)	0.62963 (7)	0.0616 (7)	
H2	0.022044	1.000078	0.646538	0.074*	
C4	0.1537 (3)	0.9336 (4)	0.56990 (7)	0.0700 (9)	
H4	0.134770	0.943043	0.546016	0.084*	
C3	0.0559 (3)	0.9828 (5)	0.59462 (7)	0.0816 (11)	
H3	−0.029809	1.025672	0.587528	0.098*	
H2O1	0.142 (3)	0.457 (2)	0.5273 (8)	0.057 (9)*	
H1O1	0.151 (4)	0.589 (3)	0.5157 (8)	0.085 (13)*	

### Atomic displacement parameters (Å^2^)

**Table:**

	*U*^11^	*U*^22^	*U*^33^	*U*^12^	*U*^13^	*U*^23^
Co1	0.0358 (2)	0.0358 (2)	0.0263 (3)	0.000	0.000	0.000
Cl1	0.1200 (10)	0.1498 (13)	0.0220 (4)	0.0660 (9)	0.000	0.000
Cl2	0.1157 (11)	0.0514 (6)	0.1035 (10)	−0.0010 (6)	0.000	0.000
N2	0.0373 (12)	0.0359 (12)	0.0249 (10)	0.0001 (9)	0.000	0.000
N1	0.0424 (10)	0.0422 (10)	0.0274 (8)	0.0000 (7)	−0.0015 (7)	0.0003 (7)
O1	0.0732 (16)	0.0850 (19)	0.0814 (16)	−0.0034 (14)	0.0074 (13)	−0.0058 (15)
C6	0.0405 (11)	0.0418 (11)	0.0282 (9)	0.0017 (8)	−0.0022 (8)	−0.0011 (8)
C5	0.0408 (11)	0.0490 (12)	0.0292 (10)	0.0040 (9)	−0.0027 (8)	−0.0004 (8)
C1	0.0541 (14)	0.0557 (14)	0.0292 (10)	0.0014 (11)	0.0042 (9)	−0.0005 (9)
C7	0.0536 (14)	0.0648 (15)	0.0281 (10)	0.0148 (11)	−0.0063 (9)	−0.0012 (9)
C8	0.065 (2)	0.069 (2)	0.0210 (13)	0.0163 (17)	0.000	0.000
C2	0.0535 (15)	0.085 (2)	0.0462 (14)	0.0173 (14)	0.0114 (11)	−0.0033 (13)
C4	0.0591 (17)	0.116 (3)	0.0346 (12)	0.0347 (17)	−0.0058 (11)	−0.0014 (14)
C3	0.0580 (17)	0.135 (3)	0.0519 (16)	0.0430 (19)	−0.0035 (13)	−0.0036 (17)

### Geometric parameters (Å, º)

**Table:**

Co1—N2^i^	1.888 (2)	C6—C7	1.385 (3)
Co1—N2	1.888 (2)	C6—C5	1.471 (3)
Co1—N1	2.0591 (18)	C5—C4	1.368 (3)
Co1—N1^i^	2.0591 (18)	C1—C2	1.361 (4)
Co1—N1^ii^	2.0591 (18)	C1—H1	0.9300
Co1—N1^iii^	2.0591 (18)	C7—C8	1.376 (3)
Cl1—C8	1.728 (3)	C7—H7	0.9300
N2—C6	1.344 (2)	C8—C7^iii^	1.376 (3)
N2—C6^iii^	1.344 (2)	C2—C3	1.370 (4)
N1—C1	1.341 (3)	C2—H2	0.9300
N1—C5	1.355 (3)	C4—C3	1.385 (4)
O1—H2O1	0.824 (18)	C4—H4	0.9300
O1—H1O1	0.843 (18)	C3—H3	0.9300
			
N2^i^—Co1—N2	180.0	N2—C6—C5	113.33 (18)
N2^i^—Co1—N1	99.69 (5)	C7—C6—C5	125.58 (19)
N2—Co1—N1	80.31 (5)	N1—C5—C4	121.8 (2)
N2^i^—Co1—N1^i^	80.31 (5)	N1—C5—C6	113.71 (18)
N2—Co1—N1^i^	99.69 (5)	C4—C5—C6	124.48 (19)
N1—Co1—N1^i^	91.623 (15)	N1—C1—C2	122.3 (2)
N2^i^—Co1—N1^ii^	80.31 (5)	N1—C1—H1	118.8
N2—Co1—N1^ii^	99.69 (5)	C2—C1—H1	118.8
N1—Co1—N1^ii^	91.623 (15)	C8—C7—C6	117.3 (2)
N1^i^—Co1—N1^ii^	160.62 (9)	C8—C7—H7	121.3
N2^i^—Co1—N1^iii^	99.69 (5)	C6—C7—H7	121.3
N2—Co1—N1^iii^	80.31 (5)	C7^iii^—C8—C7	122.3 (3)
N1—Co1—N1^iii^	160.62 (9)	C7^iii^—C8—Cl1	118.85 (14)
N1^i^—Co1—N1^iii^	91.623 (16)	C7—C8—Cl1	118.86 (14)
N1^ii^—Co1—N1^iii^	91.623 (15)	C1—C2—C3	119.3 (2)
C6—N2—C6^iii^	120.8 (2)	C1—C2—H2	120.4
C6—N2—Co1	119.59 (12)	C3—C2—H2	120.4
C6^iii^—N2—Co1	119.60 (12)	C5—C4—C3	118.7 (2)
C1—N1—C5	118.49 (19)	C5—C4—H4	120.6
C1—N1—Co1	128.45 (15)	C3—C4—H4	120.6
C5—N1—Co1	113.02 (14)	C2—C3—C4	119.4 (3)
H2O1—O1—H1O1	103 (3)	C2—C3—H3	120.3
N2—C6—C7	121.1 (2)	C4—C3—H3	120.3
			
N1—Co1—N2—C6	0.56 (12)	N2—C6—C5—N1	2.1 (3)
N1^i^—Co1—N2—C6	−89.44 (12)	C7—C6—C5—N1	−178.2 (2)
N1^ii^—Co1—N2—C6	90.56 (12)	N2—C6—C5—C4	−177.1 (3)
N1^iii^—Co1—N2—C6	−179.44 (12)	C7—C6—C5—C4	2.6 (4)
N1—Co1—N2—C6^iii^	−179.44 (12)	C5—N1—C1—C2	0.7 (4)
N1^i^—Co1—N2—C6^iii^	90.56 (12)	Co1—N1—C1—C2	−176.8 (2)
N1^ii^—Co1—N2—C6^iii^	−89.44 (12)	N2—C6—C7—C8	2.5 (3)
N1^iii^—Co1—N2—C6^iii^	0.56 (12)	C5—C6—C7—C8	−177.14 (19)
C6^iii^—N2—C6—C7	−1.31 (17)	C6—C7—C8—C7^iii^	−1.23 (15)
Co1—N2—C6—C7	178.69 (16)	C6—C7—C8—Cl1	178.78 (15)
C6^iii^—N2—C6—C5	178.4 (2)	N1—C1—C2—C3	−0.7 (5)
Co1—N2—C6—C5	−1.6 (2)	N1—C5—C4—C3	−0.1 (5)
C1—N1—C5—C4	−0.4 (4)	C6—C5—C4—C3	179.0 (3)
Co1—N1—C5—C4	177.5 (2)	C1—C2—C3—C4	0.2 (6)
C1—N1—C5—C6	−179.6 (2)	C5—C4—C3—C2	0.1 (6)
Co1—N1—C5—C6	−1.7 (2)		

Symmetry codes: (i) *y*−1/4, −*x*+5/4, −*z*+5/4; (ii) −*y*+5/4, *x*+1/4, −*z*+5/4; (iii) −*x*+1, −*y*+3/2, *z*.

### Hydrogen-bond geometry (Å, º)

**Table:**

*D*—H···*A*	*D*—H	H···*A*	*D*···*A*	*D*—H···*A*
O1—H2*O*1···Cl2	0.82	2.35	3.1735	177
O1—H1*O*1···Cl2^iv^	0.84	2.43	3.2607	170
C7—H7···O1^v^	0.93	2.44	3.334 (4)	161

Symmetry codes: (iv) −*x*, −*y*+1, −*z*+1; (v) *x*, *y*+1/2, −*z*+1.
