# Supplementary material for: Crystal structure and photoluminescent properties of bis­(4′-chloro-2,2′:6′,2′′-terpyrid­yl)cobalt(II) dichloride tetra­hydrate
Source: Acta Crystallogr E Crystallogr Commun. 2020 Mar 5;76(Pt 4):496–9. doi: 10.1107/S205698902000287X (PMC7133030; doi:10.1107/S205698902000287X)
Supplement: Supplementary file 3 [file e-76-00496-sup3.docx]

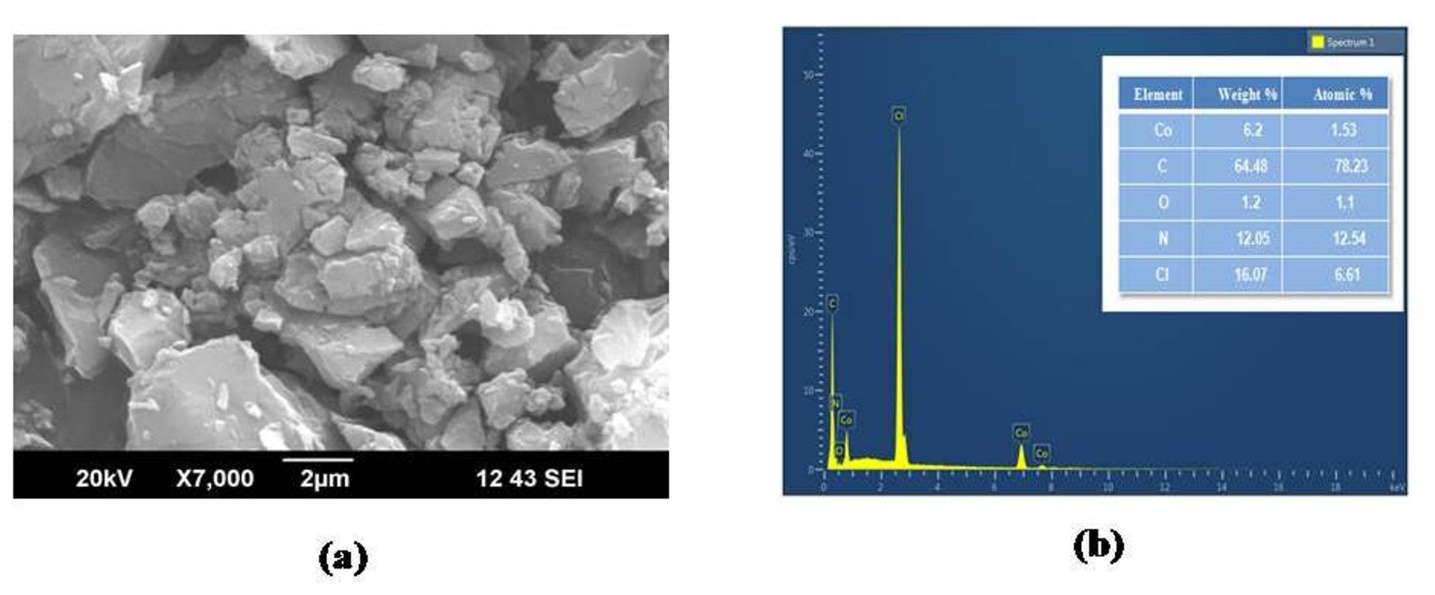


**Figure** (**a**) SEM image (**b**) EDS spectrum of the complex (**3**) and **e**lemental composition of the complex (inset table).
